# Harnessing the cGAS-STING pathway to potentiate radiation therapy: current approaches and future directions

**DOI:** 10.3389/fphar.2024.1383000

**Published:** 2024-04-10

**Authors:** Nicholas W. Colangelo, Naamit K. Gerber, Ralph E. Vatner, Benjamin T. Cooper

**Affiliations:** ^1^ Department of Radiation Oncology, NYU Grossman School of Medicine, New York, NY, United States; ^2^ Department of Radiation Oncology, University of Cincinnati College of Medicine, Cincinnati, OH, United States

**Keywords:** CGAS, STING, radiation, review, immunotherapy, oncology, STING agonists, microenvironment

## Abstract

In this review, we cover the current understanding of how radiation therapy, which uses ionizing radiation to kill cancer cells, mediates an anti-tumor immune response through the cGAS-STING pathway, and how STING agonists might potentiate this. We examine how cGAS-STING signaling mediates the release of inflammatory cytokines in response to nuclear and mitochondrial DNA entering the cytoplasm. The significance of this in the context of cancer is explored, such as in response to cell-damaging therapies and genomic instability. The contribution of the immune and non-immune cells in the tumor microenvironment is considered. This review also discusses the burgeoning understanding of STING signaling that is independent of inflammatory cytokine release and the various mechanisms by which cancer cells can evade STING signaling. We review the available data on how ionizing radiation stimulates cGAS-STING signaling as well as how STING agonists may potentiate the anti-tumor immune response induced by ionizing radiation. There is also discussion of how novel radiation modalities may affect cGAS-STING signaling. We conclude with a discussion of ongoing and planned clinical trials combining radiation therapy with STING agonists, and provide insights to consider when planning future clinical trials combining these treatments.

## 1 Introduction

Radiation therapy is an integral component of cancer treatment and involves the use of ionizing radiation to destroy cancer cells; however, it can also induce significant tumor-directed immune responses (Demaria et al., 2004; Abuodeh et al., 2016). Immune activation could be used to potentiate radiotherapy, but unfortunately this process is not well understood (Ngwa et al., 2018). In this context, the cGAS-STING pathway has gained increasing attention, as it has been shown to promote an immune response to the DNA damaging agents used to treat cancer (Yum et al., 2020). The cGas-STING pathway is not only an important cellular defense mechanism against intracellular pathogens, generating pro-inflammatory cytokines in response to pathogen-derived cytosolic double-stranded DNA (dsDNA) (Kato et al., 2017), it is now appreciated for its role in several processes, including senescence, genomic instability, autophagy, and relevant to this review, the immunosurveillance of cancer (Beernaert and Parkes, 2023).

**FIGURE 1:**
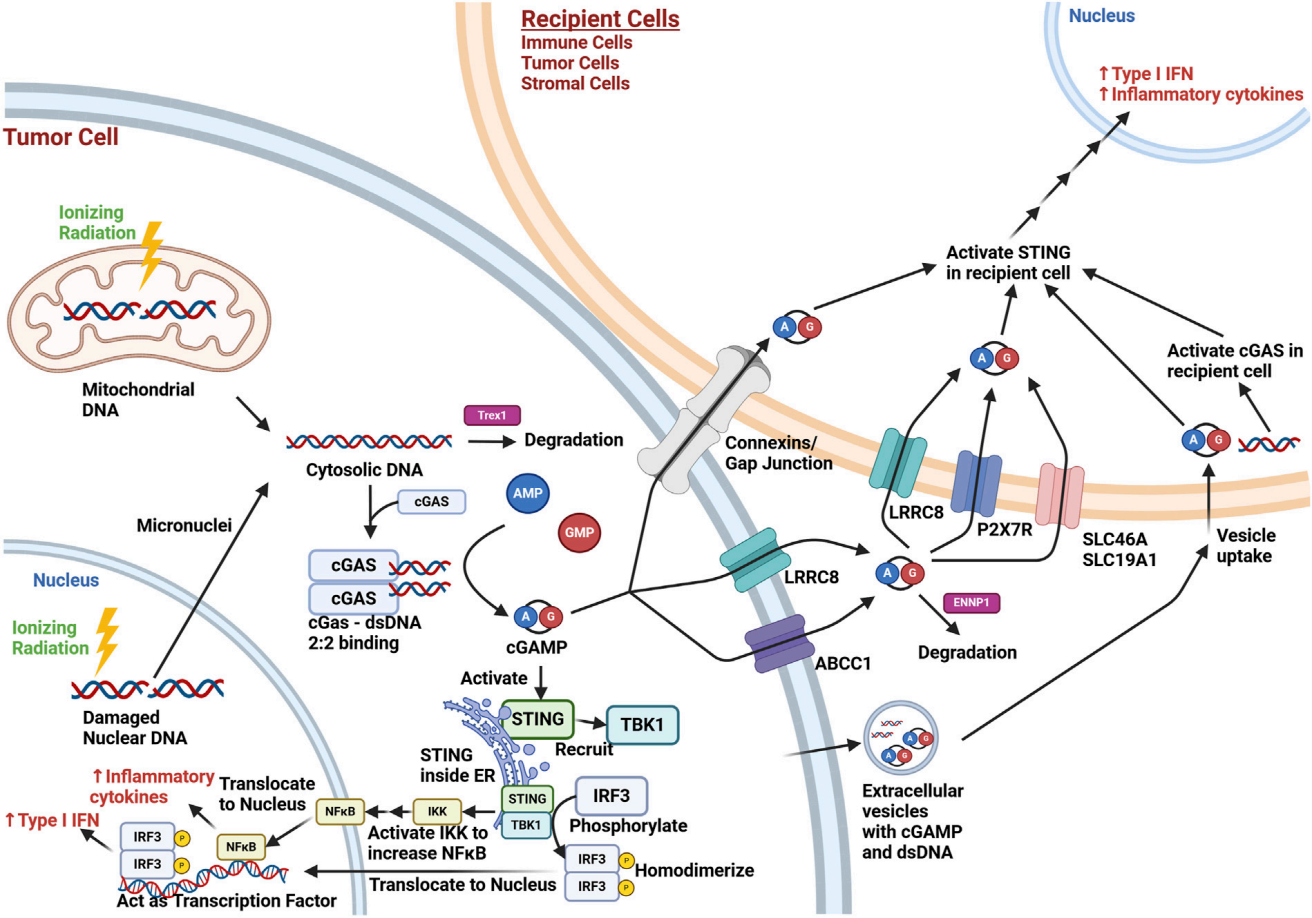
Overview of cGAS-STING Signaling in the Tumor Microenvironment after Ionizing Radiation Exposure. Ionizing radiation (lightning bolt) results in cytoplasmic release of mitochondrial dsDNA immediately and nuclear dsDNA eventually through micronuclei formation during cell division. This cytosolic dsDNA may be degraded by TREX1, a cytoplasmic endonuclease. cGAS activation involves two molecules of dsDNA bind to a dimer of cGAS (2:2 binding), which allows cGAS to form cGAMP from AMP and GMP. cGAMP intracellularly can activate STING in the endoplasmic reticulum (ER), which can then recruit TBK1. This complex brings in and phosphorylates IRF3, which then homodimerizes and translocates to the nucleus, where it acts as a transcription factor for the production of type I interferons. STING can also act through the NF-κB pathway to lead to increased inflammatory cytokine production (e.g., TNF-α, IL-1, IL-6, IL-8). cGAMP may also exit the cell via connexins/gap junctions, membrane transporters (e.g., LRCC8, ABCC1), or extracellular vesicles. dsDNA may also be contained in extracellular vesicles. Extracellular cGAMP not contained in vesicles is exposed to possible degradation by ENNP1, an endonuclease. cGAMP may be taken up by cells in the microenvironment via connexins/gap junctions, membrane transporters (LRRC8, P2x7R, SCL46A, SCL19A1), or via uptake of extracellular vesicles. The cGAMP can activate STING in recipient cells, and dsDNA uptake can lead to STING activation through recipient cell cGAS activation. This results in the paracrine/autocrine production of type I interferons and inflammatory cytokines in the tumor microenvironment. While the irradiated cell in this schematic was set to be a tumor cell, other irradiated cells in the tumor microenvironment (e.g., immune and stromal cells) may undergo similar cGAS-STING pathway signaling. Created with BioRender.com.

To discuss the role of cGAS-STING signaling in the tumor microenvironment, it is important to understand the overall pathway (Figure 1). Cyclic GMP-AMP synthase (cGAS) is a nucleotidyltransferase activated by binding dsDNA (Gao et al., 2013). cGAS has been shown to be predominantly localized to the plasma membrane in the resting state (Barnett et al., 2019), although there is also evidence for localization free in the cytoplasm, around micronuclei, or within nuclei in response to stress signals, like DNA damage (Liu H. et al., 2022). It combines guanine monophosphate (GMP) and adenosine monophosphate (AMP) to form cyclic GMP-AMP (cGAMP) (Sun et al., 2013), bound at the 3′ OH of AMP and 5′ phosphate of GMP at one end, and the 2′ OH of GMP and 5’ phosphate of AMP at the other end (Ablasser et al., 2013). Specifically, cGAS activation involves two strands of dsDNA binding with two cGAS proteins (Li et al., 2013; Zhang et al., 2014). cGAS can bind to RNA and single stranded DNA, but it is considered to be only significantly activated by dsDNA greater than around 45 base pairs, and is actually inhibited by dsDNA in the approximately 20–40 base pair range. (Chen et al., 2016; Liu et al., 2023b). The binding of cGAS is independent of dsDNA sequence, as it is due to the interactions between the protein and the sugar-phosphate backbone of dsDNA, but not the nitrogen base (Chen et al., 2016; Kato et al., 2017). However, the degree of cGAS activation upon binding DNA appears to be governed by the specific DNA sequence, its length, and any damage, due to the influence these factors can have on the mechanical flexibility of dsDNA, important for the optimal sensing of dsDNA by cGAS (Wang et al., 2022).

The cGAMP produced by cGAS activates an endoplasmic reticulum protein called stimulator of interferon genes (STING) (Wu et al., 2013; Shang et al., 2019). The conformational change induced by cGAMP binding results in STING oligomerization, with migration towards the Golgi apparatus (Dobbs et al., 2015; Ergun et al., 2019). STING recruits TANK Binding Kinase 1 (TBK1), which leads to the phosphorylation of interferon regulatory factor 3 (IRF3) (Ishikawa et al., 2009; Zhao et al., 2016; Zhao et al., 2019). This allows IRF3 to homodimerize, translocate to the nucleus, and act as a transcription factor (Tanaka and Chen, 2012), promoting type I interferon (interferon α/β) production (Yum et al., 2021). TBK1 also leads to increased nuclear factor kappa-light-chain-enhancer of activated B cells (NF-κB) signaling via activating the inhibitor of NF-κB (IκB) kinase (IKK) complex, which can phosphorylate IκB family proteins, releasing NF-κB for downstream signaling (Yum et al., 2021). As a nuclear transcription factor, NF-κB also increases inflammatory cytokines (e.g., TNF-α, IL-1, IL-6, IL-8) (Hoesel and Schmid, 2013). The activation of both IRF3 and NF-κB transcription factors results in a synergistic increase in downstream activation, making cGAS-STING a potent pro-inflammatory response pathway (Panne et al., 2007).

In this review, we will cover the effects of cGAS-STING signaling in the tumor microenvironment, with a focus on its role in the response of tumors to radiation therapy. We will also examine the current and future potential for drugs targeting the cGAS-STING pathway to potentiate radiation therapy.

## 2 Immune modulating effects of the cGAS-STING pathway in cancer

Multiple studies have helped clarify the role of the cGAS-STING pathway in the tumor microenvironment. Most of the focus has been on cancer cells and immune cells; however, this pathway should be relatively agnostic to cell type (Motwani et al., 2019). For cells in the tumor microenvironment, DNA damaging treatments (Yang et al., 2017), such as chemotherapy or radiation, can generate dsDNA fragments, which can become cytosolic dsDNA. Cancer cells can also generate cytosolic dsDNA through genomic instability (Bakhoum et al., 2018). The sensing of this cytosolic dsDNA by cGAS leads to cGAMP production, which can have several different fates ultimately leading to the production of type I interferon in the tumor. For instance, cGAMP can activate STING signaling directly within tumor cells, leading to an inflammatory response through production of type 1 interferon (Kumar et al., 2023).

However, interferon production can also occur through STING activation in other cells in the tumor microenvironment (Mekers et al., 2022). One mechanism for this is the secretion of cGAMP, where it can act as a paracrine signal, activating STING in recipient cells. For example, cGAMP can be secreted and taken up by volume-regulated anion channels, such as the leucine-rich repeat containing protein (LRRC8) (Lahey et al., 2020; Zhou et al., 2020; Concepcion et al., 2022). ATP binding cassette subfamily C member 1 (ABCC1), known for contributing to chemotherapy resistance by transporting chemotherapeutics out of cancer cells, also has been shown to export cGAMP (Maltbaek et al., 2022). This cGAMP can be then be taken up by cells with the plasma membrane transporters solute carrier family 19 member 1 (SLC19A1) (Luteijn et al., 2019; Ritchie et al., 2019) or solute carrier family 46 (SLC46A) (Cordova et al., 2021), with species and cell type specificity. The purinergic P2X7 receptor (P2X7R), a ligand-gated ion channel, has also been shown to have a role in cGAMP uptake, though it is not clear if this is direct or through its association with the formation of transmembrane channels via pannexins (Iglesias et al., 2008). Others have shown that connexin proteins can play a role in the intercellular transfer of cGAMP(Schadt et al., 2019; Pépin et al., 2020). Connexins can form hemi-channels on the plasma membrane capable of secreting contents into the extracellular space as well as connect cells for direct transfer of molecules. Both functions may contribute to cGAMP transport. Extracellular vesicles are membrane bound particles secreted by cells either directly from the plasma membrane or by fusion of internal compartments with the plasma membrane, and have been shown to carry cGAMP(Tkach et al., 2022). It has also been reported that dsDNA is contained in extracellular vesicles, which may contribute to the cGAS-STING activation in recipient cells (Diamond et al., 2018). Similarly, activated STING has been shown to be released in the extracellular vesicles of tumor cells stimulated with a STING agonist, and these extracellular vesicles were shown to promote anti-tumor immunity in mouse tumor models (Gao et al., 2022). Extracellular cGAMP is degraded by the phosphodiesterase activity of ectonucleotide pyrophosphatase/phosphodiesterase 1 (ENPP1), limiting its effectiveness as a paracrine signal for an immune response (Li et al., 2014; Kato et al., 2018; Li J. et al., 2021). It has also been demonstrated that dying cancer cells release dsDNA that can be taken up by phagocytic cells in the tumor, leading to cGAS/STING activation (Klarquist et al., 2014).

Whether immune cells are recipients of tumor-derived cGAMP or have their cGAS directly activated, both pathways result in activation of STING leading to downstream IRF3 signaling. This activation of IRF3 by STING leads to type I interferon production, which mediates an important immune signaling response in the microenvironment. Type 1 interferon has been shown to mediate an anti-cancer immune response through signaling on the interferon α/β receptor 1 (IFNAR1) on dendritic cells (Diamond et al., 2011). Specifically, the subset of dendritic cells that are CD8α^+^ appears to be important for mediating this effect (Fuertes et al., 2011), as these cells perform antigen cross presentation and priming of CD8^+^ T-cells, which are responsible for the cytotoxic anti-tumor immune response (Woo et al., 2014). Interestingly, there is evidence that *acute* type I interferon responses lead to these immunostimulatory responses, while *persistent* signaling can lead to a suppressive phenotype through immune exhaustion (Boukhaled et al., 2021). It is also important to remember that the immune response from cGAS-STING signaling may not be only due to cytokines and interferon signaling for a cytotoxic T-cell response. For instance, lymphoma cells have been shown to increase expression of retinoic acid early transcript 1 (RAE1) ligands in response to cGAS-STING signaling from cytosolic dsDNA, which targets them for elimination by NK cells (Lam et al., 2014). Considering the relative contribution of the different cellular subsets in the tumor microenvironment, it should be noted that the CD45^+^ immune cells, specifically the dendritic cell subset, have been shown to contribute to most of the type I interferon production in tumors (Deng et al., 2014; Schadt et al., 2019).

However, non-immune elements in the tumor microenvironment may also influence the immune response. In one model, endothelial cells were shown to be important in responding to cGAMP in the tumor microenvironment by producing interferon-β(Demaria et al., 2015). Similarly, cancer-associated fibroblasts (CAFs) can have STING activation leading to interferon release, contributing to the immune response (Arwert et al., 2020). The significance of CAFs is difficult to assess, as it has been shown that tumor-promoting CAFs(Ma et al., 2022) and tumor-suppressive CAFs(Kabashima et al., 2022) can be induced from cGAS-STING signaling in the cancer cells, while others have shown that a STING agonist can contribute to downregulation of CAFs as part of the immune response (Hajiabadi et al., 2023). Non-cellular elements in the tumor can also participate in the immune response. For example, the extracellular matrix has been shown to signal for an immunosuppressive phenotype in tumor cells via mechanotransduction from stress fibers leading to autophagic degradation of cGAS (Liu et al., 2023c).

It is important to recognize that cancer cells can circumvent cGAS-STING signaling and the subsequent immune response through a number of mechanisms. A study evaluating The Cancer Genome Atlas (TCGA) and other human tumor genome databases revealed that tumors frequently contain loss-of-function mutations as well as epigenetic silencing of cGAS and STING (Konno et al., 2018). For example, loss of liver kinase B1 (LKB1) was shown in the context of KRAS-driven lung cancers to silence STING expression (Kitajima et al., 2019). At the protein level, post-translational modifications, such as ubiquitination, acetylation, phosphorylation, and SUMOylation, can also modify the activity of STING and cGAS (Liu J. et al., 2022). Even mutations in tumor protein 53 (p53) can block STING signaling (Ghosh et al., 2021). Another mechanism of suppressing STING signaling is upregulation of three prime repair exonuclease 1 (TREX1), an enzyme that can prevent cGAS activation by degrading cytosolic dsDNA (Vanpouille-Box et al., 2017). Persistent cGAS-STING signaling, as happens in cancers with chromosomal instability, can lead to decreased interferon release via reduced STING levels as a form of tachyphylaxis (Li et al., 2023). STING activation has also been shown to increase PD-L1 expression in cervical cancer cells through the NF-κB pathway, which could be clinically meaningful if this translated into improved immune evasion, and would suggest a benefit for pharmaceuticals targeting the STING pathway to be given with PD-L1 inhibitors (Cai et al., 2020). There is also evidence that cancer cells can respond to the type I interferon in their environment, leading to resistance to cytotoxic T-cells via inhibition of granzyme B (Chen et al., 2019) and increased PD-L1 levels (Jacquelot et al., 2019).

Taken together, these interactions demonstrate the complex interplay between cGAS-STING signaling and the tumor microenvironment, as well as the significance of these factors when considering pharmacologic interventions.

## 3 Immune system independent effects of the cGAS STING pathway in cancer

In addition to its central role in the immune response to cancer, STING signaling has been implicated in processes independent of its immunostimulatory effect. For example, the type I interferons released as a result of STING signaling may elicit a pro-apoptotic and anti-proliferative autocrine/paracrine response in some cancer cells. Studies of exogenous administration of type I interferons *in vitro* have demonstrated that these effects of interferons are variable based on cancer cell origin, mutations, and interferon type (Rodríguez-Villanueva and McDonnell, 1995; Chawla-Sarkar et al., 2001). There is also evidence for more direct STING induction of apoptosis through IRF3 activation leading to loss of mitochondrial outer membrane permeability (Zierhut et al., 2019). Cancer cells may in part develop resistance to interferon-induced apoptosis through mutations in the apoptotic pathways downstream of the interferon-α,β receptor (Chawla-Sarkar, 2003). Interferons have also been shown to act as a pro-survival signal for some cancer cells, providing resistance to chemotherapy (Gaston et al., 2016) and radiation (Khodarev et al., 2004) *in vitro*. These findings suggest that while interferon signaling can induce apoptosis in sensitive cancer cells, apoptosis-resistant cancer cells may develop pro-survival signaling through signal transducer and activator of transcription 1 (STAT1) or other pathways. Other cell death and signaling pathways may be involved as well, although the bulk of the literature focuses on how interferon signaling affects apoptosis. For instance, colon cancer cells have been shown to undergo necroptosis in response to autocrine/paracrine interferon resulting from cGAS-STING signaling (Chen et al., 2018). Ultimately, the biological relevance of these direct effects of interferons on cancer cells is not clear, as there appears to be variability in the extent to which cancer cells express interferon receptors (Yano et al., 1999).

The activation of STING in cancer cells may also affect the survival response independent of downstream interferon production. For instance, the cGAS-STING pathway can activate autophagy, which can reduce cytosolic dsDNA and promote cell survival (Gui et al., 2019). However, STING signaling was also found to result in ferroptotic cell death in pancreatic cancer cells in an interferon-independent manner (Li C. et al., 2021). Cheradame et al. found increased DNA damage by the comet assay and decreased clonogenic survival of breast cancer cells to mafosfamide or radiation in the context of STING knockdown (Cheradame et al., 2021). This was independent of silencing IFNAR1, suggesting it is independent of interferon signaling. Knockdown of STING in multiple patient-derived xenografts resulted in reduced cell viability even in the absence of genotoxic stress. In contrast to these findings, Haymen et al. found that STING knockout decreased damage by comet assay and increased clonogenic survival of prostate cancer cells to cisplatin or radiation (Hayman et al., 2021). They demonstrated in nude mice that prostate cancer cells with STING knockout injected subcutaneously were more resistant to ionizing radiation, as assessed by tumor growth delay. The use of nude mice suggests this was independent of T cells. When attempting to reconcile these apparently contradictory studies, several factors should be considered. First, the use of different cell types can lead to different conclusions. Similarly, the difference between performing a gene knockdown or knock-out is significant, as signaling pathways can be sensitive to the difference between reduced *versus* no signaling. Lastly, Cheradame et al. found their effect was independent of paracrine/autocrine interferon signaling, while Hayman et al. noted that interferon-stimulated gene 15 levels were increased in response to radiation in the wild-type prostate cancer cells, but abrogated in the STING knockout cells, suggesting that the pro-survival effect of STING knockout they observed may be mediated by the tumor cells responding to STING-mediated interferon production.

Overall, this section emphasizes the challenges inherent in attempts to manipulate these signaling pathways for therapeutic benefit: The net effect of cancer cell intrinsic responses as well as tumor microenvironment and immune responses must be considered when evaluating whether a therapeutic intervention is expected to be beneficial.

## 4 Ionizing radiation and the cGAS STING pathway

Radiation therapy to treat cancer uses ionizing radiation to slow or prevent the growth and spread of tumors as well as microscopic neoplastic disease. According to classical radiobiology, the principal mechanism is through direct and indirect damage to genomic DNA in the nucleus causing preferential damage and killing of cancer cells; however, ionizing radiation also activates numerous signaling pathways and genetic programs in both neoplastic and non-neoplastic cells (Joiner and van der Kogel, 2018). Although the exact mechanism is a current area of investigation, one such byproduct of cellular irradiation is the generation of cytosolic dsDNA, which can be from nuclear (Mackenzie et al., 2017) or mitochondrial (Yamazaki et al., 2020) origin. The relative importance of these two sources of cytoplasmic dsDNA in initiating radiation-induced inflammation is an area of active research and controversy. Although nuclear dsDNA and micronuclei appear to correlate with radiation-induced inflammation, it may take days for this to stimulate a response, since mitotic progression is an important component of micronuclei formation (Harding et al., 2017). In contrast, mitochondrial dsDNA enters the cytosol within hours after irradiation (Patrushev et al., 2006), and therefore may potentially be more important for initial cGAS activation. Cytoplasmic dsDNA generated by radiation may be an especially potent inducer of cGAS signaling due to the oxidative damage and modifications caused by ionizing radiation (Goodhead, 1989), potentially making it more resistant to cytosolic nucleases like TREX1 (Gehrke et al., 2013). The resultant cGAMP leads to an inflammatory response that is thought to have a role in radiation-induced inflammation and the abscopal effect, wherein radiation treatment at one site of disease causes shrinking of distant sites via the immune system (Galluzzi et al., 2023).

In preclinical models, the anti-tumor immune effect of radiation appears dependent on STING activation leading to type I interferon production in dendritic cells. This can occur from cGAMP produced by cGAS directly in dendritic cells (Deng et al., 2014) or via cGAMP secreted from tumor cells (Schadt et al., 2019). The cGAMP released from cancer cells can be degraded by ENPP1 present in the microenvironment, limiting the relative contribution of cGAMP from the tumor cells (Carozza et al., 2020a). This may in part explain the difference in Deng et al. and Schadt et al. regarding the relative importance of cGAMP derived from cancer cells *versus* dendritic cells in mediating an anti-tumor immune response. dsDNA released by the tumor cells can also be taken up by dendritic cells, resulting in cGAS activation (Diamond et al., 2018; Fang et al., 2021). It has been shown that canonical NF-κB signaling in dendritic cells is important for the STING-dependent anti-tumor immunity seen with ionizing radiation, while non-canonical NF-κB signaling was found to be inhibitory (Hou et al., 2018). It was demonstrated *in vivo* that a non-canonical NF-κB pathway inhibitor could produce tumor growth delay with 20 Gy ionizing radiation in an interferon-dependent manner using an immunocompetent mouse with subcutaneous tumors (Hou et al., 2018). These results support the role of dendritic cells in promoting anti-tumor immunity in response to ionizing radiation. Interestingly, one study found that radiation exposure to normal tissue, not the tumor microenvironment, can still elicit a significant antitumor effect in a p53-dependent manner, although the exact mechanism was not clarified (Camphausen et al., 2003). Given the role of STING signaling in normal tissue inflammation and immune surveillance in response to ionizing radiation (Dou et al., 2017), it is reasonable to consider if cGAS-STING signaling is involved. Moreover, the p53 dependence in the host mice that was necessary for the anti-tumor effect in Camphausen et al. is consistent with recent findings that p53 signals for TREX1 degradation, resulting in cytoplasmic dsDNA accumulation, cGAS-STING pathway activation, and ultimately immune-dependent tumor regression (Ghosh et al., 2021; Ghosh et al., 2023).

There is debate about the optimal radiation dose and fractionation for stimulating an anti-tumor immune response stemming from apparently conflicting observations in different studies. Pivotal work by Vanpouille-Box et al. provided a mechanistic underpinning for the observation that moderate hypofractionation (e.g., 8 Gy x three fractions) is more effective at inducing a systemic anti-tumor response than treatment with a larger dose of 20 Gy delivered in a single fraction (Vanpouille-Box et al., 2017). Using a syngeneic TSA breast cancer model, they demonstrated that a higher 20 Gy radiation dose had a reduced immune response relative to multiple fractions of lower doses (e.g., 8 Gy x three fractions) due to reduced secretion of type I interferon. This was attributed to the induction of TREX1, a cytosolic endonuclease that degraded dsDNA in the cytosol, preventing cGAS activation. The improved response to multiple fractions of lower doses relative to a single high dose fraction was corroborated by Yamazaki et al., who demonstrated that autophagy-competent cancer cells could avoid this response through enhanced clearing of cytosolic dsDNA (Yamazaki et al., 2020). More specifically, it appears autophagy can clear micronuclei (Bartsch et al., 2017) and damaged mitochondria (Lindqvist et al., 2018) before they can release dsDNA into the cytosol. However, it should be noted that studies exist showing that higher doses (e.g., over 20 Gy) can generate an interferon response in a tumor model, though without CTLA-4 blockade (Burnette et al., 2011; Filatenkov et al., 2015). These discordant observations suggest there is additional nuance to be explored in this area; perhaps that there may be significant variation in the threshold dose for TREX1 activation in different cancers. What has been consistent, however, is that dendritic cells and the interferon receptor IFNAR1 are important for mediating the cytotoxic T-cell immune response in mouse models (Burnette et al., 2011; Vanpouille-Box et al., 2017). Collectively, these results emphasize that details of the radiation treatment (e.g., dose and fractionation) as well as the cancer cells (e.g., autophagy, TREX1 activation) are important considerations for evaluating radiation-induced immunogenic responses.

There are potential limitations to using radiation to stimulate cGAS-STING. Radiation generates significant oxidative stress, which can activate the nuclear factor erythroid 2-related factor 2 (NRF2) pathway. NRF2 is a transcription factor that is released from inhibition in response to oxidative stress. While this pathway can increase the levels of reactive oxygen species scavengers, helping to reduce damage mediated from oxidative stress, it has been shown to suppress STING expression, thereby reducing interferon signaling (Olagnier et al., 2018; Jessen et al., 2020). The nuances of how the transcription factor achieves this effect have not yet been clarified. However, this suggests that in cancer cells that do not already have defective STING signaling, oxidative stress from chemotherapy or radiation treatment could lead to reduced STING signaling. It would be valuable for potential clinical applications to clarify whether this is a transient or persistent effect in response to NRF2 signaling, as it may inform the timing of STING agonists relative to these treatments. Cancer cells exposed to ionizing radiation have also been shown to use caspases to reduce their type 1 interferon production as well as the tumor-directed immune response *in vivo* (Rodriguez-Ruiz et al., 2019; Han et al., 2020). To the extent that interferon production from cancer cells is significant, these pathways represent potential barriers to the radiation-induced interferon response. Other effects of radiation include the observation that irradiated mesenchymal stem cells can promote tumor metastasis in a mouse lung metastasis model, which could be blocked by knocking down the cGAS-STING pathway in the irradiated mesenchymal stem cells (Zheng et al., 2020). Radiation may also activate inflammatory cGAS-STING in normal non-immune cells, which may lead to normal tissue toxicity via pyropotosis; however, it was demonstrated in an intestinal model that ultrahigh dose rates may spare normal tissue from this effect (Shi et al., 2022).

## 5 Preclinical results for radiation therapy with STING agonists

A variety of STING agonists have been developed to promote an immunogenic response through STING activation (Hines et al., 2023). This has led several groups to test whether STING agonists could be used to promote the immunogenic response seen with radiation treatment (Table 1). *In vitro*, intratumoral administration of cGAMP results in enhanced tumor regression with increased interferon-related T-cell response (Deng et al., 2014). Synthetic cGAMP analogues were created to circumvent limitations associated with direct cGAMP administration, such as improving stability and efficacy (Motedayen Aval et al., 2020). Even before the flavone acetic acid derivative called 5,6-dimethylxanthenone-4-acetic acid (DMXAA) was known to be a STING agonist, it was understood to elicit anti-cancer activity through anti-vascular and anti-immune effects (Daei Farshchi Adli et al., 2018), and demonstrated synergy with ionizing radiation (Pedley et al., 1996; Wilson et al., 1998). Eventually, DMXAA was shown to be a STING agonist capable of significant immune-related tumor regression *in vivo* (Corrales et al., 2015), although it is notable for several disappointing clinical trials (Hines et al., 2023), attributed to its binding of STING in mice, but not humans (Conlon et al., 2013). ADU-S100, a synthetic cyclic dinucleotide (CDN), was shown to have an effect on tumor size but did not appear to have significant synergy with radiation treatment in a rat model of esophageal adenocarcinoma, comparing tumor size before radiation treatment and 8 weeks afterwards (Zaidi et al., 2021). Interestingly, only the groups receiving ADU-S100 prevented relative tumor growth at 8 weeks, and the radiation treatment alone produced no tumor growth delay. Without more information about the tumor growth kinetics or a description of the radiation technique, it is difficult to draw definitive conclusions about this finding. Another type of STING agonist, a small molecule dimeric amidobenzimidazole (diABZI), was shown to work with radiation in cultured non-small cell lung cancer cells, with the combination reducing clonogenic survival through increased apoptosis (Xue et al., 2022). A synthetic phosphodiesterase-resistant cGAMP derivative was injected concurrently and 24h after mice received 10 Gy in one fraction, causing an early TNFα dependent response from the tumor microenvironment followed by T-cell mediated tumor regression, with control of distant, non-irradiated tumors (Baird et al., 2016). This early TNFα effect mediated by the tumor microenvironment has also been shown for STING agonists in the absence of radiation treatment (Francica et al., 2018), indicating the importance of understanding how STING agonists affect normal tissue in order to optimize clinical implementation.

**TABLE 1:** STING agonist and ionizing radiation pre-clinical studies.

Study	Tumor cells	Model	STING agonist	Dose	Admin	Timing	RT dose and freq	Major outcome
Pedley et al. (1996)	Human LS174T colon adenocarcinoma	Nude female mice with subcutaneous flank tumors	DMXAA	27.5 mg/kg	IP	48h after RT	18.5MBq of I131 tagged to anti-CEA antibody injected via tail vein once	Synergistic decrease in tumor volume with 83% complete tumor regression, persistent until termination at 1 year
Wilson et al. (1998)	Mouse MDAH-MCa-4 mammary tumors, RIF-1 fibrosarcoma	Female C3H/HeN with intramuscular tumors	DMXAA	−80 μmol/kg (single fraction RT) −56 μmol/kg (fractionated RT)	IP	5min after RT (after every other RT for fractionated)	20Gy x 1 2.5Gy x 8 (BID)	Synergistic effect on tumor growth delay with single RT dose, but additive with fractionated RT regimen
Deng et al. (2014)	Mouse MC38 colorectal cancer	C57BL/6J mice with subcutaneous flank tumor	2′3′-cGAMP	10 μg	IT	Day 2 and 6 after RT	20Gy x 1	Synergistic decrease in tumor volume with 70% tumor rejection for RT + cGAMP, lost in STING deficient mice
Baird et al. (2016)	Mouse Panc02 pancreatic adenocarcinoma	C57BL/6 mice with subcutaneous flank tumors	Phosphodiesterase resistant cGAMP-derivative	1, 10, 25 μg	IT	Immediately after RT and again 24h after RT.	10Gy x 1	Synergistic decrease in tumor size at 10 μg. Immune-mediated decrease in size of distant implanted tumor
Zaidi et al. (2021)	N/A	Sprague-Dawley rats with acid reflux induced esophageal adenocarcinoma	Synthetic CDN ADU-S100	50 μg	IT	At time of RT and 3 weeks after RT.	16Gy x 1	No synergy between ADU-S100+RT comparing tumor size pre-RT vs8 weeks post-RT
Xue et al. (2022)	Human H460 and A549 NSCLC cells	Cell culture	Small molecule agonist diABZI	20 nM	M	2 h before RT	2Gy x 1 4Gy x 1	diABZI + RT had additive effects on inhibiting cell proliferation and clonogenic survival, with promotion of apoptosis
Liu et al. (2019)	Mouse B16F10-OVA melanoma and 4T1 breast cancer	C57BL/6 mice with IV injection of B16F10-OVA or mammary fat pad with 4T1 to form lung metastases	Phosphatidylserine-coated liposome loaded with cGAMP	37 μM	INH	24h after each RT (3 doses total)	8Gy x 3 daily to right lung only	Synergistic immune-dependent decrease in lung metastases with STING agonist + RT. “Cured” mice with 4T1 model resisted rechallenge
Zhang et al. (2023)	Mouse CT-2A glioma	C57BL/6 mice with injected brain tumors	Small molecule agonist diABZI in nanoparticles	0.25 mg/kg diABZI	ICC or IV	ICC: Last day of RT and 4 days after RT (2 doses total) IV: Day after RT, then day 6 and 11 after RT (3 doses total)	3Gy x 3 daily	diABZI-NP + RT had synergistic improvement in survival whether by ICC or IV. “Cured” mice resisted rechallenge with CT-2A tumors
Luo et al. (2019)	Mouse B16F10-OVA melanoma and TC-1 HPV-induced lung cancer	C57BL/6 mice with subcutaneous flank tumors	PEG-b-PC7A based micelles with peptide antigen (piece of OVA for B16F10 and of E7 protein for TC-1)	30 μg	SC	Immediately after RT and 7 days post RT	20Gy x 1	Synergistic effect of RT + STING agonist on local tumor growth as well as distant tumor growth. For local tumors, 50% of mice tumor-free at 60 days post-RT with combination, and these mice could resist re-challenge
Luo et al. (2022)	Mouse CT26 and MC38 colorectal cancers	C57BL/6 for MC38 and BALB/c for CT26 with subcutaneous flank tumors	cGAMP-MOL	2 μg cGAMP 0.5 μmol MOL	IT	1 day before RT start	2Gy x 6 daily	cGAMP-MOL synergized with RT for tumor growth inhibition. “Cured” mice with CT26 tumors resisted rechallenge
Wang et al. (2021)	Mouse B16F10 melanoma and CT26 colorectal cancer	Female C57BL/6 for B16F10 and female BALB/c for CT26 with subcutaneous flank tumors	Mn-Alginate	100 nmol Mn (∼5.5 μg)	IT	1 day after 1st RT dose	5Gy x 2 (CT26) 8Gy x 2 (B16F10) (Doses 2 days apart)	Mn-Alginate + RT reduced volume of irradiated and distant tumors, and improved survival, relative to RT alone
Yan et al. (2022a)	Mouse 4T1 mammary carcinoma	Female BALB/c mice with mammary fat pad tumors	Mn-MPN	1.3 mg/kg Mn	IV	Day before each RT (3 doses total)	6Gy x 3 (Every other day)	Mn-MPN had at least additive effects with RT on tumor growth of primary and distant tumors
Carozza et al. (2020a)	Mouse 4T-1 breast cancer	Female BALB/c mice with mammary fat pad tumor	ENPP1 inhibitor SFT-1084 and cGAMP	100 nM SFT-1084 and 10 μg cGAMP	IT	Dose at day 2, 4, 7 post-RT (3 doses total)	20Gy x 1	STF-1084 synergized with RT and cGAMP to delay tumor progression
Baird et al. (2018)	Mouse Panc02-SIY pancreatic adenocarcinoma	C57BL/6 mice with flank tumor	ENPP1 inhibitor MV-626	60 mg/kg	IP	1 dose day before RT, 1 current with RT, then 3 daily doses post RT	10Gy x 1 15Gy x 1 20Gy x 1	Found 6 of 6 mice eliminated tumor with MV-626 and 20 Gy, and 4 of those resisted re-challenge. 10Gy and 15Gy had less dramatic responses with MV-626. Note: Poster-presentation
Jin et al. (2023)	Mouse MOC2 head and neck squamous cell carcinoma and B78 melanoma	Female C57BL/6 mice with subcutaneous flank tumors	ATM inhibitor AZD0156	10 mg/kg	PO	1 h before RT then daily for 4 days after RT (5 doses total)	8Gy x 1 (MOC2) 12Gy x 1 (B78)	AZD0156+RT had synergistic effects on primary and distant MOC2 tumors*
Liu et al. (2023a)	Mouse MC38 and CT26 colorectal cancer	Female C57/B6J for MC38 and BALB/c for CT26 mice with subcutaneous flank tumors	ATR inhibitor berzosertib	60 mg/kg	PO	2 h before RT and daily for 3 days after RT (4 doses total)	5Gy x 1	Berzosertib + RT had at least additive effect on tumor size*

List of preclinical studies combining a STING agonist with ionizing radiation. Abbreviations: Admin, Route of Administration; CDN, cyclic dinucleotide; cGAMP-MOL , cGAMP metal-organic layer; Mn , Manganese; NSCLC, non-small cell lung cancer; MPN, Metal-phenolic network; IP, intraperitoneal; IT, intratumoral; IV, intravenous; ICC, intracranial cannula; INH, inhalation with nebulizer; SC, subcutaneous injection; M , added to medium; PO, oral; RT, radiation treatment.

^a^ Note: ATM/ATR inhibitors interfere with DNA damage repair and may mediate their STING activation by interfering with the repair of radiation-induced DNA damage resulting in increased cytoplasmic DNA.

Other pre-clinical studies have explored using nanoparticles containing cGAMP, with the idea that nanoparticles can accumulate in tumors and stabilized cGAMP(Motedayen Aval et al., 2020). Inhalable nanoparticles with cGAMP were able to synergize with radiation in a mouse model of lung metastases through enhanced antigen presenting cell and T-cell activation, leading to synergistic anti-tumor responses (Liu et al., 2019). diABZI was also tagged to CD47/PD-L1 targeting nanoparticles for intratumoral injection into a mouse glioblastoma model, leading to improved T cell infiltration in the brain and synergy with radiation delivered as 9 Gy in three fractions, using survival as the endpoint (Zhang et al., 2023). A STING-activating nanoparticle-based vaccine had STING-dependent synergy with radiation treatments both on the primary irradiated tumor as well as in implanted distant tumors, using a subcutaneous mouse flank model (Luo et al., 2019). Another group used a nanoscale metal-organic layer (MOL) impregnated with cGAMP to take advantage of the radiosensitizing properties of the MOL in addition to its ability to retain cGAMP in the tumor microenvironment (Luo et al., 2022). Intratumoral injection of cGAMP-MOL in a mouse model led to improved STING activation and regression in tumors irradiated with 12 Gy in six fractions.

Manganese has also been shown to activate STING, however, it is difficult to localize to the tumor. Several attempts have been made to overcome this using intratumorally injected scaffolds. Wang et al. demonstrated that intratumorally injected alginate with manganese leads to increased radiation-induced immunity using 10 Gy in two fractions (Wang et al., 2021), while Yan et al. used a metal-phenolic network to coordinate intratumoral administration of a radiationsensitizer (high-Z material) with manganese, and demonstrated tumor growth inhibition with ionizing radiation delivered as 18 Gy in three fractions (Yan J. et al., 2022).

Another way to promote the inflammatory response downstream of STING would be to use small molecule inhibitors of ENPP1, which can degrade extracellular cGAMP (Carozza et al., 2020a; Carozza et al., 2020b). This approach is significant as we gain more appreciation of the role of ectonucleotidases in reducing the effectiveness of cancer treatments (Stagg et al., 2023). A poster presentation provided preliminary results showing that the orally administered ENPP1 inhibitor, MV-626, acted synergistically with radiation treatment, resulting in tumor eradication in the six mice tested (Baird et al., 2018). Drugs targeting both ataxia telangiectasia mutated (ATM) and ataxia telangiectasia mutated and Rad3 related (ATR) proteins, involved in DNA damage repair, were administered with ionizing radiation in mouse tumor models and demonstrated increased CD8^+^ T-cell infiltration through canonical and non-canonical STING activation (Liu C. et al., 2023; Jin et al., 2023). This effect is presumably mediated through increased cytosolic dsDNA produced by blocking these DNA repair process proteins.

These studies collectively demonstrate the potential for STING agonists with radiation therapy. However, there needs to be caution when extrapolating preclinical results, especially in mice, to what could be expected in clinical trials. Similarly, the radiation doses in these studies were mostly large, single fractions of radiation. Notably, the studies using the largest single doses, such as 20 Gy in one fraction, seemed to find synergy between radiation and STING agonists, whereas the studies using lower doses often found just additive effects. This would align with the results of Vanpouille-Box et al., discussed previously, who found that larger single doses of radiation resulted in TREX1 activation, leading to degradation of cytoplasmic DNA and a reduced tumor-directed immune response (Vanpouille-Box et al., 2017). It may be that STING agonists could be a way to overcome the effects of this potential TREX1 activation at high doses. This observation is also important to emphasize because these large doses are not comparable to current and historical standards of radiation therapy. Radiation treatments are typically delivered in multiple smaller doses (e.g., 2 Gy per fraction), although larger fraction sizes can be used in stereotactic body radiotherapy (SBRT). These findings suggest that the role of STING agonists may be more meaningful in the context of SBRT, but need additional preclinical studies to more intensely evaluate the role of radiation dose and fractionation. Regarding the timing of STING agonists relative to the radiation treatment, it is difficult to draw any definitive conclusions. However, it may be that delivering STING agonists only before radiation treatment, and not after, is associated with additive responses. This aspect of the timing should be tested directly in preclinical studies, as it is relevant to future clinical trials. Another consideration is that several of these studies used tumor cells modified to express tumor-specific antigens, such as OVA and SIY, or were HPV-induced, all of which will lead to the stronger potential for an immune response. In these studies, there remains the question of whether the responses would still be as potent for a more immunologically cold tumor. However, many studies discussed did not use these, and still demonstrated that synergistic responses were possible. Despite these limitations, these studies can help guide future preclinical works, as well as provide some early insights for clinical trial design.

## 6 Considerations for clinical trials of radiation therapy with STING agonists

At this time, the formula for significant anti-tumor immune responses from radiation treatment remains elusive (Galluzzi et al., 2023). Findings from early phase clinical trials suggest that immune modifying agents might support radiation-induced abscopal effects (Golden et al., 2015; Welsh et al., 2019; Curti et al., 2020). However, local radiation treatments leading to distant tumor response in the clinic remain largely anecdotal (Abuodeh et al., 2016; Dagoglu et al., 2019). STING agonists are one approach to amplifying this radiation-related immune response. Currently, there are no available clinical results regarding the efficacy of STING agonists with radiation therapy, but herein we will evaluate the currently available clinical and preclinical information in the literature.

A synthetic cyclic dinucleotide (CDN) analog called TAK-676 (eazostinag disodium) is a well-characterized and potent STING agonist (Carideo Cunniff et al., 2022). In contrast to many other CDN-based drugs that utilize intratumor delivery, limiting treatment to accessible tumors, TAK-676 can be delivered intravenously. This is being used in a Phase I trial of patients with advanced non-small cell lung cancer, triple negative breast cancer, or squamous cell head and neck cancers who progressed on checkpoint inhibitor treatment and have two or more lesions. This trial will have one lesion treated with radiation therapy as 24 Gy in three fractions, followed by PD-1 inhibitor on day 1 and dose-escalated TAK-676 at day 1, 8, 15 (Cooper et al., 2022). Although the primary endpoint of the trial is safety, the secondary endpoints of tumor response at the irradiated site as well as at the non-irradiated lesions will provide early insights into the resultant anti-tumor immune response from this regimen.

Given what we reviewed so far, optimal STING agonist trial design should consider the role of radiation dose and fractionation, as high doses may activate TREX1, hampering any natural STING signaling through cGAMP generation (Vanpouille-Box et al., 2017). Further, the timing and dose of STING agonists may be important, as persistent STING agonism may lead to immune cell apoptosis, inadvertently decreasing immune responses. For instance, T-cells can respond to STING agonism with apoptosis, leading to a dampened immune response to tumors (Gulen et al., 2017). It appears that calibrated, lower levels of STING activation are needed to promote immunogenicity and avoid this effect (Sivick et al., 2018). Preclinical work shows that cancer cells can develop compensatory mechanisms to resist the effects of STING agonists (Lemos et al., 2020). As mentioned in the previous section, radiation activates the NRF2 response, which can lead to decreased STING expression and poorer responses to STING agonists (Olagnier et al., 2018). STING also appears to have a role in DNA damage repair, and it remains to be clarified whether adding STING agonists to radiation treatment would be protective or sensitizing to cancer cells in this respect. The timing of STING agonist administration relative to radiation treatment may also be important. It is clear that additional preclinical data are needed to help clarify the optimal timing, frequency, and dosing of STING agonists, as well as the radiation treatments.

The effect of dose rate on STING signaling is another area of practical importance, particularly in the context of low dose rate brachytherapy and targeted radionuclide therapy. Unfortunately, there is limited evidence in the literature regarding the effects of low dose rates on STING signaling. Patel et al. were able to show that yttrium-90 (^90^Y), a β-particle emitter with a 2.7 days half life, could be preferentially targeted to tumors by tagging it to an alkylphosphocholine called NM600 (Patel et al., 2021). When combined with immune checkpoint inhibitors (ICIs), this radionuclide resulted in complete responses in 45%–66% of mice using an immunologically cold syngeneic tumor cell line. Notably, this effect was STING dependent. This group also published a comparison of this ^90^Y-tagged NM600 with standard external beam radiation therapy (EBRT) in a mouse model (Jagodinsky et al., 2021). Using their syngeneic mouse model, they demonstrated that type 1 interferon release was similar between the two approaches, but the increase was delayed with the use of the radionuclide relative to EBRT. This delay may be due to the low dose rate, requiring more time to cause enough damage to stimulate cGAS-STING signaling. Interestingly, they also found in their mouse model that the CD8^+^ T cells had increased type 2 interferon release, but that it was unaffected by knockout of STING in the tumor cells. This suggests that cGAS-STING signaling in the microenvironment, or even a STING-independent mechanism in the tumor or its microenvironment, may be contributing to the observed intratumoral immune response. Yang et al. explored the role of STING signaling at low dose rates using radium-223 (^223^Ra), an α-particle emitter with an 11.43 days half-life that targets to bone, in a mouse model with syngeneic tumor cells in the bone marrow (Yang et al., 2024). They found that STING knockout in the tumor cells increased tumor cell viability and allowed greater tumor growth in response to ^223^Ra treatment. They attributed this difference to the tumor cells with STING knockout or antagonism having reduced pyroptosis. The immunomodulatory effects of these low dose rates treatments will be important to help optimize radionuclide treatments, particularly as lutetium-177, a β-particle emitter with a 6.6 days half-life, is gaining more clinical applications (Jia et al., 2022; Constanzo et al., 2023; Fallah et al., 2023). In the context of this review, if radionuclides could be tagged to STING agonists, then their combined effects could contribute to increased efficacy through enhanced tumor-directed immune responses. However, additional preclinical studies are needed in this area to help clarify the role of STING signaling using low dose rate radiotherapy techniques.

There are preclinical findings that could be considered when attempting to improve clinical efficacy. For instance, loading CDNs in extracellular vesicles has been demonstrated in mouse tumor models to improve the effectiveness of CDNs(Jang et al., 2021). A Phase I/II clinical trial (NCT04592484) involving intratumor injection of CDNs loaded into extracellular vesicles called exoSTING for advanced/metastatic solid tumors, by Codiak Bioscience, had a press release mentioning signs of clinical efficacy, but the study has been halted with no published results to date (Ducrot et al., 2023). These results, even if negative, will be helpful in guiding the field towards optimal drug delivery.

Novel radiation delivery techniques may also lead to synergy with STING agonists through differential effects on the immune system relative to conventionally delivered radiation therapy. FLASH radiotherapy and lattice/grid therapy both allow higher doses of radiation to be delivered while avoiding normal tissue toxicity (Bertho et al., 2023). FLASH delivers ultrahigh dose rates (>40 Gy/s compared to 2 Gy/min with standard radiotherapy) of ionizing radiation, which through mechanisms not fully understood, leads to preferential sparing of the normal tissue (Limoli and Vozenin, 2023). For instance, in a preclinical model, delivering radiation at ultrahigh dose rates was able to avoid the toxicity from inflammation caused by STING activation in the normal tissue that was seen with conventional dose rates, while preserving efficacy against the tumor (Shi et al., 2022). Lattice therapy and its counterpart GRID deliver spatially-separated high doses of radiation within a tumor such that the normal tissue, including the intratumoral lymphatic and immune structures, have reduced toxicity. This potential preservation of immune cells in the microenvironment, while still damaging tumors, has been shown in at least one preclinical model to lead to immune effects even in distant, unirradiated tumors (Kanagavelu et al., 2014), and there is at least one case report demonstrating efficacy against a large (63cc) tumor combined with PD-1 immunotherapy (Jiang et al., 2021). Personalized ultrafractionated stereotactic adaptive radiation therapy (PULSAR) delivers larger doses of radiation spaced farther apart in time, to capitalize on tumor shrinking and changes in the tumor microenvironment. PULSAR was used in mouse tumor models with PD-L1 antibodies to demonstrate a synergistic response of radioimmunotherapy (Moore et al., 2021). They also demonstrated that when delivering two fractions of 15 Gy spaced over 10 days, delivery of PD-L1 with the second fraction, but not the first, was important for the anti-tumor immune response. These findings lead to questions about whether the addition of STING agonists to this regimen could further promote an immunogenic response. There are currently two active clinical trials looking to combine PULSAR treatment with the STING agonist IMSA101 (NCT05846659, NCT05846646). Lastly, pre-clinical evidence suggests that carbon ion irradiation of cancer cells produce similar immune responses through the STING pathway relative to x-rays (Du et al., 2021). However, the signaling may be stronger with carbon ions (Guo et al., 2023), possibly related to their enhanced radiobiological effectiveness (Schlaff et al., 2014). Ultimately, the radiobiology of these novel radiation modalities and delivery techniques is still under investigation, and the effect of these on tumor control and immune activation are active research questions, complicating studies using these techniques in combination with pharmacological immune activators.

There should also be some consideration for the potential normal tissue toxicity from STING agonists. While current trials seem to demonstrate mild toxicity (Hines et al., 2023), preclinical data suggests the potential for STING-related injury. For instance, cGAS-STING can signal for pyroptosis in normal tissues (Yan M. et al., 2022; Zhang et al., 2022), and this has been demonstrated to play a role in intestinal toxicity to ionizing radiation exposure through pyroptosis (Shi et al., 2022). Similarly, cGAS-STING signaling in macrophages can also lead to necroptosis through proinflammatory cytokine production, and the STING agonist DMXAA was shown to result in sterile shock in mice, which was not present when administered to STING deficient mice (Brault et al., 2018). These finding suggest that cytokines induced from STING agonists could cause a cytokine release syndrome (Khadka et al., 2019). Methods of targeting STING agonists to tumors (Su et al., 2019; Wu et al., 2022), or simply having tocilizumab available (Le et al., 2018), may be meaningful clinically to prevent potential systemic inflammatory responses.

## 7 Conclusion

In this review, we have discussed the significance of cGAS-STING signaling in the context of radiation therapy. We covered its activation by genotoxic stress resulting in cytosolic dsDNA. The various modes of intercellular signaling mediated by cGAMP and the role of STING activation in cancer cells and in immune cells were considered. The immune stimulating *versus* immunosuppressive potential was explored, including the role of shorter exposures to STING agonists to avoid immunosuppression. We covered the complexity of STING signaling in the DNA damage response, both in tumor cells and their microenvironment. We applied these ideas specifically to the cellular response to radiation, exploring the nuances of the pathways involved. We discussed the promising results of combining radiation treatments with STING agonists in preclinical studies, reviewed ongoing studies combining STING agonists and radiation, and provided considerations for how these may influence study design for future clinical trials. We also evaluated the potential role for new radiation treatment technologies, and how they may capitalize on cGAS-STING signaling. We look forward to future works clarifying the complex interplay of cGAS-STING signaling in cancer cells and their microenvironment, and the immense potential for combining STING agonists with radiation therapy.
